# Flanged scleral fixation: thermoplastic properties of suture materials and implications for flange geometry

**DOI:** 10.1186/s12886-026-05091-2

**Published:** 2026-07-03

**Authors:** Melih Parlak, Jens Ulrich Werner, Muhammet Cinar, Armin Wolf

**Affiliations:** Department of Ophthalmology, Ulm University, Prittwitzstr. 43, 89075 Ulm, Germany

**Keywords:** Flange, Intraocular lens, Scleral fixation, Suture

## Abstract

**Background:**

Flanged scleral fixation techniques are increasingly used in the management of intraocular lens dislocation and aphakia. However, complications such as conjunctival erosion and scleral migration remain a concern. Flange geometry may play a critical role in anchoring stability. This study investigates the thermoplastic properties of various suture materials to identify optimal conditions for flange formation.

**Methods:**

A disposable electrocautery device with a power supply and tip temperature of 392 °C was used for standardised application. Seven suture materials were used: polypropylene (6/0), polyamide 6 (6/0), polyamide 6.6 (6/0), vinylidene fluoride-co-hexafluoropropylene (PVDF) (6/0), polyester (5/0), and polytetrafluoroethylene (PTFE) (5/0 and 6/0). All sutures were heated 0.5, 1, 2, 3, 4, and 5 mm from the distal end and examined in 4 repeat measurements. The prepared flanges were photographed and measured using a digital microscope. The flange dimensions and geometry were assessed. Ratios of flange width to flange length and flange width to suture diameter were calculated.

**Results:**

All suture materials except polyamide 6.6 (6/0) and PTFE (5/0) formed a flange shape due to the increase in temperature as demonstrated by repeated measurements with a high degree of reproducibility. While polypropylene and PVDF took on a mushroom/rhomboid shape, the flange shape of polyamide 6 and polyester was spherical. The flange geometry of PTFE was funnel-shaped with sharp edges. With increasing duration of heat exposure, the flange length of polypropylene and PVDF increased more significantly than the flange width.

**Conclusion:**

Polypropylene and PVDF demonstrate favorable thermoplastic properties for flanged scleral fixation, enabling reproducible flange formation with short heating times. A heating length of approximately 1 mm appears optimal for controlled flange geometry. Further biomechanical and in vivo studies are required to validate clinical performance.

## Background

In a multitude of surgical contexts, the human sclera serves as a stabilising anchor point for traumatically altered irises or dislocated intraocular lenses. Conventionally, fine suture materials were utilised for this purpose; however, these materials have been observed to pose significant risks, including suture breakage and conjunctival erosion [1, 2].

Alternatively, flanged scleral fixation can be considered, aiming to be more resistant and durable due to its higher suture strength. Over the past decade, several flanged fixation techniques have been introduced, including the Yamane double-needle technique, the Canabrava double-flanged fixation method, and modified double-flanged approaches for iris, capsular tension ring, and intraocular lens fixation [3, 4]. These techniques share the principle of creating a thermally induced flange that acts as an anchoring element within the sclera or adjacent tissue. While flanged scleral fixation is gaining increasing attention for various surgical indications, complications such as scleral migration, conjunctival erosion, flange exposure, intraocular lens tilt, and even endophthalmitis have also been described [5, 6].

The flange size and geometry are crucial for secure anchoring. This study examines the thermoplastic properties of suture materials to identify the material with the most suitable in vitro thermoplastic properties and the optimal heated suture length for flanged scleral fixation while acknowledging that biomechanical performance was not directly assessed.

## Methods

For standardised use, a disposable electrocautery (Bovie Medical, Florida/USA) was operated with a power supply unit that provided the same voltage as the battery originally supplied (I = 3.23 A). A thermocouple was used to measure the temperature at the tip of the electrocautery at distances of 1, 1.5 and 2 mm. All examinations were carried out using a surgical microscope and a calibration ruler. The cautery tip was positioned at a constant distance of approximately 1 mm from the suture material without direct contact. The heating process was terminated when the melted segment reached the grasping point of the forceps. The duration of heat application was recorded with a digital stopwatch.

A total of seven types of suture material were utilised in the study: polypropylene (6/0; Prolene, Ethicon), vinylidene fluoride-co-hexafluoropropylene (PVDF) (6/0; Pronova, Ethicon), polyamide (5/0; Supramid, Serag-Wiessner), polyamide 6.6 (6/0; Ethilon, Ethicon), multifilament polyester (5/0, Ethibond Excel, Ethicon), and polytetrafluoroethylene (PTFE) (5/0 and 6/0; Seramon, Serag-Wiessner).

All sutures were grasped 0.5, 1, 2, 3, 4, and 5 mm from the distal end using the same type of microforceps under microscopic visualisation. Care was taken to maintain a consistent grasping position and pressure throughout all experiments. The free-standing suture segment was then melted up to the forceps by applying heat from a defined distance (1 mm). The experiment was repeated four times for each suture material and heating length. The prepared sutures were photographed and measured using a digital microscope (Keyence VHX-7000, Keyence Corp, Osaka/Japan). Image analysis and dimensional measurements were performed manually using the integrated microscope software. Measurements were repeated independently to minimise observer-dependent variation. The flange length, flange width, flange geometry, and heating time were examined.

Statistics: All data were analysed descriptively, dependent on their scale level, using Microsoft Excel and R for statistical computing (version 4.3.2). For all continuous variables the arithmetic mean, standard deviation (SD), and range were calculated. Graphical representation was based on line charts. A two-way analysis of variance (ANOVA) was conducted, with suture material type and heating length designated as the two independent variables. Independent Welch t-tests were performed to compare flange width, flange length and heating time between Prolene (polypropylene) and Pronova (PVDF) for each heating length.

## Results

The cautery tip was found to have a temperature of 392 °C. At distances of 1 mm, 1.5 mm, and 2 mm, the temperatures were recorded as 84 °C, 44 °C, and 28 °C, respectively. Consequently, a distance of ca.1 mm was maintained during the heating process.

All but two suture materials formed a flange shape due to the increase in temperature. In the case of polyamide 6.6 (Ethilon), asymmetric and irregular melting was observed. Despite a melting time of over 10 s, no flange formed with 5/0 polytetrafluoroethylene (PTFE; Seramon). Consequently, these sutures were excluded from the microscopic measurements, as no flange, defined as a widening of the suture material, was observed.

**Fig. 1:**
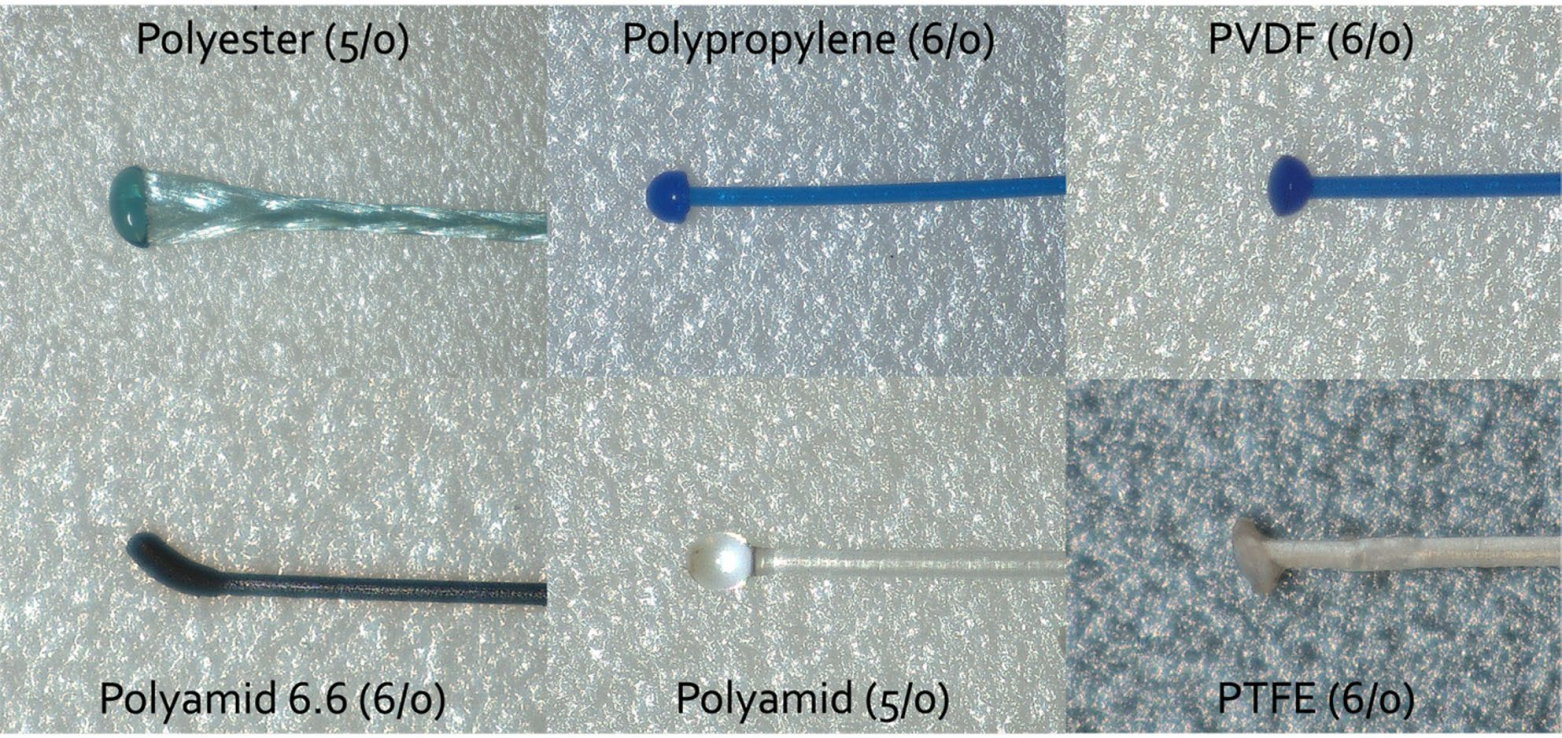
Flange shape of the tested sutures with a heating length of 1 mm. (PVDF: vinylidene fluoride-co-hexafluoropropylene; PTFE: polytetrafluoroethylene)

**Fig. 2:**
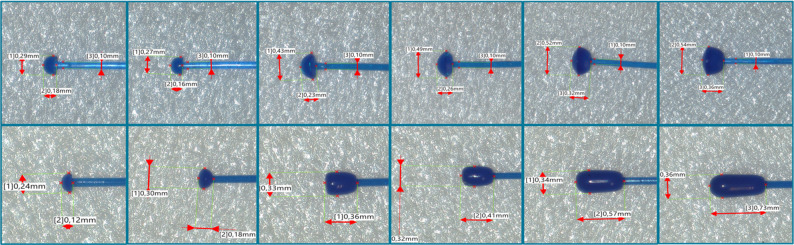
Change in flange geometry with increasing heating length for polypropylene and vinylidene fluoride-co-hexafluoropropylene (PVDF), (from left to right: 0.5, 1, 2, 3, 4, 5 mm), (Top row: polypropylene; bottom row: PVDF)

The shape of the flange varied significantly depending on the type of suture and the heating length (Figs. 1 and 2). The quantitative flange parameters differed significantly according to suture material and heating length, with significant interaction effects for all measured outcome variables (all *p* < 0.001) (Table 1).

**Table 1:** Effects of suture material and heating length on flange-related outcomes

Outcome	Material		Heating length		Interaction	
	F (df)	*p*-value	F (df)	*p*-value	F (df)	*p*-value
Flange length (mm)	F(4, 102) = 35.24	< 0.001	F(5, 102) = 107.59	< 0.001	F(18, 84) = 30.68	< 0.001
Flange width (mm)	F(4, 102) = 40.23	< 0.001	F(5, 102) = 16.51	< 0.001	F(18, 84) = 26.69	< 0.001
Flange width / length	F(4, 102) = 16.72	< 0.001	F(5, 102) = 14.52	< 0.001	F(18, 84) = 51.25	< 0.001
Flange width / suture diameter	F(4, 102) = 44.40	< 0.001	F(5, 102) = 25.89	< 0.001	F(18, 84) = 26.73	< 0.001
Heating time (s)	F(4, 103) = 130.79	< 0.001	F(5, 103) = 23.34	< 0.001	F(19, 84) = 13.95	< 0.001

Global p values were calculated using two-way ANOVA with suture material and heating length as fixed factors

By contrast, the dimensions of the flange and the application time of the cautery were highly reproducible with predominantly low coefficients of variation. Polypropylene and PVDF showed the most consistent thermoplastic behaviour with minimal inter-test variability, whereas polyester exhibited greater variability, particularly for flange width measurements at short heating lengths (Table 2).

**Table 2:** Distribution of flange dimensions in different heating lengths

Name	Material	Thickness	Suture diameter (mm)	Heating length (mm)	Heating time s (SD)	Flange length mm (SD)	Flange width mm (SD)	Flange Width/Length	Flange Width/ Suture Diameter
Prolene	Polypropylene	6/0	0.1	0.5	1.98 (0.06)	0.16 (0.01)	0.25 (0,01)	1.56	2.53
				1	1.14 (0.18)	0.20 (0.01)	0.35 (0,03)	1.51	2.98
				2	1.73 (0.19)	0.23 (0.01)	0.44 (0,01)	1.91	4.4
				3	2.67 (0.33)	0.28 (0.01)	0.51 (0,01)	1.84	5.05
				4	3.27 (0.85)	0.31 (0.01)	0.53 (0,01)	1.72	5.25
				5	3.55 (0.67)	0.35 (0.02)	0.53 (0,01)	1.51	5.25
Ethibond Excel	Polyester	5/0	0.22	0.5	§	§	§	§	§
				1	3.03 (0.14)	0.24 (0.01)	0.75 (0,09)	3.08	3.42
				2	3.93 (0.39)	0.37 (0.05)	0.47 (0,08)	1.27	2.15
				3	4.89 (0.89)	0.46 (0.01)	0.52 (0,03)	1.11	2.36
				4	4.99 (0.65)	0.51 (0.01)	0.61 (0,03)	1.2	2.78
				5	9.56 (2.61)	0.58 (0.04)	0.63 (0,03)	1.09	2.88
Pronova	Vinylidene fluoride-co-hexafluoropropylene (PVDF)	6/0	0.11	0.5	2.03 (0.29)	0.14 (0.02)	0.26 (0,03)	1.93	2.18
				1	1.92 (0.15)	0.18 (0.01)	0.31 (0,01)	1.69	2.77
				2	2.41 (0.21)	0.34 (0.02)	0.32 (0,01)	0.94	2.91
				3	2.98 (0.09)	0.45 (0.03)	0.33 (0,01)	0.74	3
				4	3.76 (0.32)	0.59 (0.01)	0.35 (0,01)	0.6	3.18
				5	4.19 (0.37)	0.69 (0.03)	0.37 (0,01)	0.54	3.39
Supramid	Polyamid	5/0	0.14	0.5	2.68 (0.32)	0.28 (0.04)	0.29 (0,03)	1.02	2.05
				1	3.19 (0.32)	0.31 (0.01)	0.31 (0,01)	1.01	2.21
				2	3.80 (0.36)	0.40 (0.01)	0.40 (0,01)	0.99	2.84
				3	6.45 (0.95)	0.47 (0.01)	0.46 (0,01)	0.98	3.3
				4	6.40 (0.19)	0.52 (0.02)	0.51 (0,02)	0.98	3.62
				5	10.59 (1.41)	0.55 (0.01)	0.55 (0,01)	1	3.93
Seramon	Polytetrafluoroethylen (PTFE)	6/0	0.14	0.5	§	§	§	§	§
				1	10.26 (0.14)	0.22 (0.01)	0.38 (0.02)	1.77	2.73
				2	10.38 (0.19)	0.33 (0.02)	0.39 (0,01)	1.2	2.81
				3	10.40 (0.19)	0.45 (0.02)	0.40 (0,01)	0.88	2.86
				4	10.28 (0.13)	0.49 (0.04)	0.40 (0,03)	0.82	2.88
				5	10.22 (0.13)	0.55 (0.03)	0.43 (0,01)	0.78	3.09

(§: There was no flange formation after heat exposure;)

**Fig. 3:**
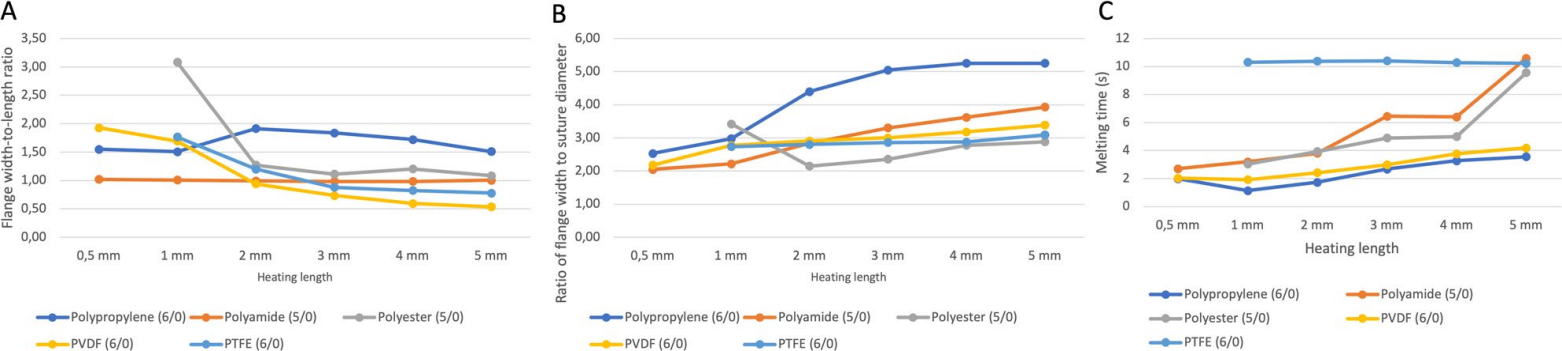
Graphical representation of the heating time and geometric indices of the flanges at various heating lengths. (**A**: Flange width-to-length ratio; **B**: Ratio of flange width to suture diameter; **C**: heating time). § No flange shape with PTFE and polyester at a heating length of 0.5 mm

Polypropylene showed a mushroom-shaped flange at heating distance of 0.5 to 2 mm, while it changed to a rhomboid shape as the heating distance increased. The heating time was relatively short, ranging between 1 and 4 s. The highest ratio between flange width and flange length was achieved with 2 mm of prepared suture material (Fig. 3).

The heating time for PVDF was similarly favourable to that for polypropylene and did not differ significantly (*p* = 0,275-0,537). However, the two filaments differed in terms of flange shape. The flange length increased more significantly than the flange width for PVDF (Table 3). The largest width-to-length ratio was achieved with a heating distance of 0.5 mm. Figure 2 shows the different flange shapes of polypropylene and PVDF depending on the heating length.

**Table 3:** Pairwise comparison of polypropylene (Prolene 6 − 0) and PVDF (Pronova 6 − 0) at identical heating lengths

Heating length (mm)	Heating time (*p*-value)	Flange length (*p*-value)	Flange width (*p*-value)
0,5	0.537	0.478	1.000
1,0	0.537	0.478	1.000
2,0	0.461	**0.004**	**< 0.001**
3,0	0.478	**0.004**	**< 0.001**
4,0	0.478	**< 0.001**	**< 0.001**
5,0	0.275	**< 0.001**	**< 0.001**

Two-sided Welch t-tests; *n* = 4 measurements per material and heating length

The flange shape for polyamide (Supramid) was spherical in all lengths of heated material and had a very smooth surface. From a heating length of 3 mm, thermal damage was observed in the form of discolouration of the flange tip and a rougher surface.

As the only multifilament suture, the heating process for polyester differed from that of monofilament sutures. When gripped with forceps, the suture split at the tip, so that short-distance heating did not produce a flange. From a heating length of 2 mm, a spherical flange with a smooth, round surface formed. The flange became larger with increasing heating length, but not wider, so that the maximum flange width was achieved with 1 mm heating.

Polytetrafluoroethylene (PTFE) was the most heat-resistant suture material. After prolonged heating, 6/0 PTFE formed a funnel-shaped flange. The width differed only slightly. All flanges were sharp-edged at the end of the suture. In contrast, even after prolonged heating, only a limited distance of 1–2 mm could be melted with 5/0 PTFE, and no flange formed.

## Discussion

The use of flanged suture material for scleral fixation is becoming increasingly important in the treatment of dislocated lenses, aphakia, and traumatic iris changes. To avoid complications such as conjunctival erosion, scleral migration, and endophthalmitis, it is crucial to achieve stable intrascleral anchoring and a practical, reproducible application method. The present study clearly demonstrates the differences in the thermoplastic properties of various suture materials.

Clinical context for the relevance of reproducible flange formation was provided by Yamane et al. in a prospective, noncomparative series of 100 eyes from 97 patients with aphakia, dislocated IOLs, or subluxated crystalline lenses. Reported complications included iris capture in 8 eyes, vitreous haemorrhage in 5 eyes, and cystoid macular oedema in 1 eye [3]. This clinical experience established the feasibility of the double-needle technique, but it also underscores that secure haptic fixation and controlled flange creation are integral to its safety profile.

Further follow-up information on suture-based flanges was provided by Canabrava and Carvalho [4] in a prospective series of 71 eyes (61 patients) treated with the double-flanged 5 − 0 polypropylene technique. Across 173 flanges and a mean follow-up of 28.2 months, 13 flanges (7.5%) were located sub-Tenon, five flanges (2.9%) became exposed at a mean of 1.8 weeks, and one patient with large flanges developed conjunctival inflammation and hyperaemia. These observations emphasise that external flange configuration and its relationship to the conjunctiva are clinically relevant, complementing the present assessment of flange shape and size.

Most recently, Icoz et al. [7] retrospectively compared patients receiving a PMMA-haptic IOL (*n* = 32) or a PVDF-haptic IOL (*n* = 28) with a modified Yamane technique. At 6 months, the groups did not differ significantly in visual acuity, refraction, intraocular pressure, or early and late postoperative complications. Intraoperative haptic damage occurred in three PMMA cases and in no PVDF cases (*p* = 0.09). Although flange dimensions were not quantified, these clinical findings support the relevance of haptic material for surgical handling and provide real-world context for the material-dependent thermoplastic differences observed in the present study.

Among the materials tested, polypropylene and PVDF in particular exhibited favourable thermal and geometric properties. Both materials formed reproducibly stable flanges with a short melting time and homogeneous shape. Polypropylene produced a mushroom-shaped flange with a broad base, which may theoretically increase tissue resistance to retraction into the vitreous cavity. The most commonly used suture material in the literature is polypropylene. However, the present study did not include biomechanical pull-out testing, and therefore no direct conclusions regarding long-term anchoring stability can be drawn.

On the other hand, PVDF exhibited a more elongated, rhomboid flange shape. The width-to-length ratio for heating lengths of 0.5 and 1 mm was similar to that of polypropylene. From a heating length of 2 mm onwards, the flange length increased significantly more than the width. This potentially allows deeper embedding in the sclera but may entail a slightly higher risk of migration. Ma et al. conducted a biomechanical study investigating the pull-out resistance of intraocular lenses (IOLs) with flanged scleral fixation [8]. The PVDF IOL haptic with a heating distance of 1 mm showed the highest pull-out resistance compared to PMMA haptics. At the same time, PVDF also exhibited the greatest flange width at the same heating length, ultimately confirming the importance of flange geometry.

Stunf Pukl et al. [9] reached the same conclusion when they compared the extraction force of flanged PVDF and PMMA haptics through human sclera. Using a 30G scleral tunnel, the extraction force of the typically mushroom-shaped PVDF flange was twice that of the funnel-shaped PMMA flange. An even more pronounced difference was observed with a 27G tunnel, where no pull-out resistance could be measured at all with the PMMA flange. This study also confirms the advantages of the mushroom-shaped flange design, which applies to both polypropylene and PVDF.

Polypropylene sutures are known to undergo long-term oxidative degradation, which has been well documented for fine-caliber 10 − 0 sutures used in scleral fixation (1,2). However, these findings cannot be directly extrapolated to the considerably thicker 5 − 0 or 6 − 0 sutures investigated in the present study, which possess a substantially larger cross-sectional area and are therefore theoretically less susceptible to complete structural failure. In contrast, PVDF demonstrates superior resistance to oxidation, fatigue, and creep owing to its highly stable fluorinated polymer backbone, while maintaining excellent biocompatibility and long-term mechanical stability [10, 11]. Nevertheless, clinical evidence comparing the long-term durability of 5 − 0 or 6 − 0 polypropylene and PVDF sutures in flanged scleral fixation remains limited, and potential material-related advantages of PVDF therefore remain largely theoretical.

Polyamide exhibited a very smooth, spherical flange shape, which, thanks to its uniform surface, is expected to be well tolerated by tissue. However, prolonged heating led to thermal damage, including discolouration, which could mean an increased risk of structural weakening or biological incompatibility.

Polyester was the only multifilament material examined. It exhibited significant variability during the melting process; a spherical flange only formed at a heating length of 2 mm or more, whereas a wide, irregular flange formed with a heating length of 1 mm. In classic suturing techniques, the advantage of polyester lies in its stable knot hold, high knot security, and flexible and pliable handling [12]. However, these properties may make it difficult to insert the suture intraocularly into a cannula. Due to this and the splicing behaviour at the forceps contact point, polyester appears to be of limited suitability for flange formation.

As expected, polytetrafluoroethylene (PTFE) proved to be highly heat-resistant, requiring more than three times the heating time of the other filaments to reach a length of 1 mm. While 6/0 PTFE formed a funnel-shaped flange after prolonged heating, 5/0 PTFE showed virtually no flange formation. The pronounced thermal resistance of this material thus limits its practical applicability for flanged scleral fixation. In addition, the sharp-edged flange shape could promote tissue erosion.

Existing evidence on flanged scleral fixation has predominantly focused on surgical outcomes rather than standardised analysis of flange geometry. Kronschlaeger et al. published a study on the melting behaviour of polypropylene that yielded results similar to those presented in this paper [13]. The clinical studies discussed above demonstrate that both material selection and flange configuration may be relevant to surgical handling and tissue-related complications [3, 4, 7]. In addition, reports on flange erosion and exposure underline the clinical relevance of reproducible flange geometry. This study is the first to provide a systematic experimental analysis of the physical properties of different suture materials with regard to flange formation. Which suture material is ideal for flanged scleral fixation and what represents the optimal heating length can only be answered cautiously on the basis of the present data, since biomechanical tensile tests and in vivo investigations were not part of this study.

Limitations of the study include the fact that only thermoplastic properties were examined in vitro. Mechanical stress tests, pull-out resistance, ageing effects, and biological tissue reactions were not evaluated. Furthermore, the experimental setup cannot fully reproduce clinical conditions such as tissue cooling, variable contact angles, intraocular manipulation, hydration of the ocular surface, or dynamic tension on the suture material during surgery. The measurements were based on manual image analysis and may therefore be subject to a certain degree of observer-dependent variability despite repeated measurements. Finally, the relatively small number of repetitions per material limits the statistical interpretation of subtle geometric differences between flange configurations.

In conclusion, polypropylene and PVDF demonstrated the most reproducible thermoplastic behaviour among the investigated materials and allowed controlled flange formation with comparatively short heating times. A heating length of approximately 1 mm appeared to provide a favourable balance between flange width and reproducibility under experimental conditions. However, the clinical relevance of these findings must be confirmed in future biomechanical and histological studies before definitive recommendations for surgical practice can be made.

## Data Availability

All data generated or analyzed during this study are included in this article. Further enquiries can be directed to the corresponding author.
